# Drug-Induced Glucose Metabolism Disorders: Role of Aryl Hydrocarbon Receptor

**DOI:** 10.3390/jox15060206

**Published:** 2025-12-02

**Authors:** Alevtina Y. Grishanova, Maria L. Perepechaeva

**Affiliations:** Institute of Molecular Biology and Biophysics, Federal Research Center of Fundamental and Translational Medicine, Timakova Str. 2, Novosibirsk 630060, Russia; aiugrishanova@frcftm.ru

**Keywords:** drug-induced disorders, aryl hydrocarbon receptor, AhR signaling, AhR ligands, glucose metabolism, insulin resistance

## Abstract

Pharmacological compounds can disrupt glucose homeostasis, leading to impaired glucose tolerance, hyperglycemia, or newly diagnosed diabetes, as well as worsening glycemic control in patients with pre-existing diabetes. Traditional risk factors alone cannot explain the rapidly growing global incidence of diabetes. Therefore, prevention of insulin resistance could represent an effective strategy. Achieving this goal requires a deeper understanding of the mechanisms underlying the development of insulin resistance, with particular attention to the aryl hydrocarbon receptor (AhR). AhR, a transcription factor functioning as a xenobiotic sensor, plays a key role in various molecular pathways regulating normal homeostasis, organogenesis, and immune function. Activated by a range of exogenous and endogenous ligands, AhR is involved in the regulation of glucose and lipid metabolism as well as insulin sensitivity. However, current findings remain contradictory regarding whether AhR activation exerts beneficial or detrimental effects. This narrative review summarizes recent studies exploring the role of the AhR pathway in insulin secretion and glucose homeostasis across different tissues, and discusses molecular mechanisms involved in this process. Considering that several drugs act as AhR ligands, the review also compares how these ligands affect metabolic pathways of glucose and lipid metabolism and insulin sensitivity, producing either positive or negative effects.

## 1. Introduction

The number of diseases caused by medicinal products has increased significantly in recent years. Drug-induced diseases associated with damage to organs and tissues [1,2,3] have become a serious public health problem. In 2017, the World Health Organization (WHO) announced its global patient safety challenge concerning medication safety [4].

Prior to market release, new drugs undergo rigorous safety evaluation; however, certain adverse drug reactions may only be identified after a medication has been used for an extended period by a large population [5]. Despite the pharmacovigilance systems established in many countries to monitor drug-induced diseases, the incidence of iatrogenic conditions remains underestimated due to the low level and quality of spontaneous reporting, as well as limitations in data availability and related factors [3,6,7].

The use of pharmacological agents can easily disrupt the balanced function of the dynamic and sensitive endocrine system. Drugs may induce endocrine disorders through various mechanisms, including alterations in hormone production, changes in the regulation and feedback of associated molecular pathways, impairment of hormone transport, and related processes [8].

Pharmacological agents may affect various components of the endocrine system. Some drugs affect the human endocrine system at the level of steroid synthesis or metabolism, mineralocorticoid receptor activity, or the catecholaminergic system, leading to elevated blood pressure [9]. Other drugs exert off-target effects on the adrenal glands [10]. The FDA recommends assessing such agents for their potential to influence steroidogenic enzymes involved in the synthesis of adrenal androgens, mineralocorticoids, and glucocorticoids [9]. Antipsychotic drugs may induce clinical manifestations of hyperprolactinemia [11]. A number of targeted anticancer therapies and immunotherapies have been associated with thyroid dysfunction [12,13]. Adverse effects of certain pharmacological agents also frequently include disturbances in glucose metabolism—namely hyperglycemia and insulin resistance—which may provoke the development of diabetes mellitus (DM) [14,15,16,17,18,19,20,21,22].

Drug-induced DM represents a serious problem in clinical practice, with a higher likelihood of development in individuals predisposed to glucose metabolism disorders due to genetic factors or unhealthy lifestyle habits, including overeating and excess body weight [23,24]. Drugs may alter glucose homeostasis by reducing tissue sensitivity to insulin, stimulating weight gain, or impairing insulin secretion. They can disrupt glucose homeostasis and provoke impaired glucose tolerance, hyperglycemia, or newly diagnosed DM, as well as worsen glycemic control in patients with pre-existing diabetes [25]. Some of these agents increase hepatic glucose production, induce acute pancreatitis, or exert direct cytotoxic effects on pancreatic β-cells [23,25]. Statins may increase the risk of diabetes by 9–33% [26].

The factors contributing to the development of diabetes are well studied, but traditional risk factors cannot fully explain the rapid increase in the global prevalence of this disease. Therefore, additional factors, such as exposure to various xenobiotics, are being studied [27].

Obesity induced by environmental toxins, insulin resistance, and diabetes development are known to be mediated by the ligand-activated transcription factor aryl hydrocarbon receptor (AhR), which functions as a xenobiotic sensor [28,29,30].

AhR is a pleiotropic transcription factor that regulates the expression of numerous genes, which may exert distinct functions in different cell types and physiological conditions. On the one hand, AhR participates in xenobiotic metabolism; on the other, it is involved in normal homeostasis, organogenesis, and immune system function. Both exogenous and endogenous AhR ligands include compounds such as polycyclic aromatic hydrocarbons (PAHs), as well as structurally diverse low-molecular-weight substances, including drugs. Upon entering the cell, ligands bind to AhR and induce the activation of signaling cascades. The AhR signaling pathway is associated with the regulation of various genes, including those related to glucose metabolism, lipid metabolism, and the development of insulin sensitivity.

A comprehensive literature search since 2014 was conducted using PubMed and Web of Science to identify studies involving AhR in drug-induced glucose metabolism disorders. The following keywords were used: glucose metabolism/tolerance, insulin resistance/sensitivity, drug effects on glucose metabolism disorders, drug induced endocrine disorders, AhR.

## 2. Glucose Metabolism and Its Disruption Induced by Xenobiotics

### 2.1. Glucose Metabolism and Homeostasis

Glucose homeostasis is primarily controlled by the liver, adipose tissue, and skeletal muscle [31]. Excess glucose is stored in the body as glycogen (mainly in the liver and muscles), while, when necessary, glucose can be generated from non-carbohydrate substrates through gluconeogenesis [32,33,34]. The principal regulators of the balance between glucose utilization and synthesis are two hormones with opposing effects—insulin and glucagon [31,32].

Insulin is produced by β-cells and released in response to increased plasma glucose and amino acid levels after food intake. During fasting or physical activity, when plasma glucose levels are reduced, glucagon is secreted by α-cells [31]. Insulin is the main physiological anabolic agent, stimulating the accumulation and synthesis of glucose, lipids, and proteins, while suppressing their breakdown and release into the bloodstream. [31,35]. Through hormone-regulated signaling cascades and depending on the organism’s energy status (deficit or surplus), either glycogen catabolism with glucose release occurs (during energy deficiency, under the action of glucagon), or glycogen synthesis with consumption of excess glucose (during energy surplus, under the action of insulin) [36,37].

There are two main categories of glucose transporters present in humans. Sodium-dependent glucose transporters (SGLTs) mediate active sodium transport across the membrane, which then diffuses along its concentration gradient together with a glucose molecule. Sodium-independent glucose transporters (GLUTs) mediate passive glucose transport across the plasma membrane [38]. GLUT1, the main glucose transporter, is present throughout all tissues of the body [39]. GLUT2 is expressed mainly in pancreatic β-cells, the liver, and the kidneys [40]. GLUT3 is present predominantly in the brain [32,40]. GLUT4 is found in the heart, skeletal muscles, adipose tissue, and the brain [40]. Among these, only GLUT4 expression is regulated by insulin [36,37]. Virtually all mammalian cells have insulin receptors (IRs), although the main tissues affected by insulin are muscle, adipose tissue, nervous tissue, and the liver [41]. The IR is a member of the receptor tyrosine kinase subfamily. It is a tetrameric protein consisting of two α- and two β-subunits. [42,43].

**Figure 1:**
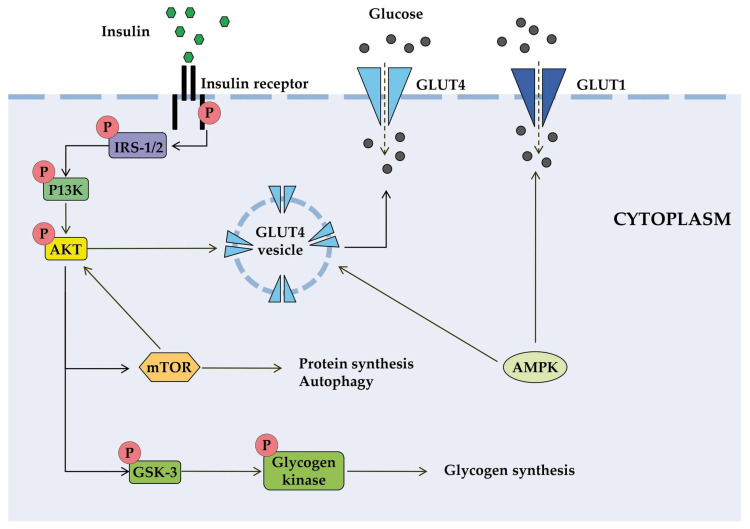
Schematic diagram of some signal transduction pathways initiated by insulin (highly simplified in this figure). Insulin binds to the α-subunit of insulin receptor, causing conformational changes that allow autophosphorylation of several tyrosine residues on the β-subunit. In the AKT signaling pathway, the phosphotyrosine-binding domains of adaptor proteins, such as members of the IRS family of insulin receptor substrates, recognize β-subunit residues of the insulin receptor, leading to phosphorylation of key tyrosine residues on IRS proteins. Activation of AKT requires PI3K, which induces phosphorylation of AKT. Once activated, AKT leads to phosphorylation and inactivation of GSK, which activates glycogen synthase. AKT also directly activates the transcription factors mTOR and Fork-head. AKT plays a significant role in the translocation of GLUT4 to the plasma membrane, resulting in glucose entry into the cell. AMPK is proposed to interact with insulin signaling and GLUT4 translocation. AKT, activates protein kinase B; GLUT, glucose transporter; GSK3, glycogen synthase kinase 3; IRS, members of the insulin receptor substrate family; mTOR, mammalian target of rapamycin; PI3K, protein kinase 3′-phosphoinositide-dependent protein kinase-1.

Insulin is involved in a complex network of interacting signaling pathways [41]. The IR initiates signal transduction upon insulin binding, leading to a cascade of intracellular events that regulate metabolism (Figure 1). The activated receptor phosphorylates insulin receptor substrates (IRS), which subsequently activate phosphoinositide-3-kinase (PI3K). This increases the level of the secondary messenger PIP3, which activates protein kinase B (AKT). AKT promotes glucose uptake through the translocation of GLUT4 transporters and facilitates glucose storage as glycogen [44,45]. Insulin signaling also affects gene expression through alternative pathways involving Ras and mitogen-activated protein kinases (MAPK) [45].

AKT regulates glucose utilization via FOXO transcription factors and mammalian target of rapamycin complex 1 (mTORC1), which control the expression of genes involved in metabolic pathways [46]. FOXO factors stimulate gluconeogenesis and negatively regulate the expression of genes that promote glucose utilization. mTORC1 regulates the expression of hypoxia-inducible factor-1 (HIF-1α) and SREBP1c, which control genes essential for glucose metabolism. mTORC1 activity itself is regulated by AKT phosphorylation [47].

Since the range of normal glucose levels is very narrow, insulin, as a regulator of its homeostasis, must respond quickly to various changes. Insulin degrades rapidly after dissociation from the receptor; the insulin signal can be terminated by internalization and degradation of IR [48]. In addition, insulin acts as a negative regulator of its own signaling [49]. Each step of the insulin signaling cascade represents a reversible enzymatic reaction. For each insulin-activated kinase, specific phosphatases exist that terminate its activity, and there is a group of phosphatases that actively suppress the action of insulin-activated kinases [41].

### 2.2. Impairment of Glucose and Lipid Metabolism and Diabetes Mellitus

Disruption of glucose homeostasis results from impaired insulin secretion or action, leading to DM—a metabolic disorder characterized by elevated blood glucose levels. The global prevalence of DM has increased significantly over the past several decades and is predicted to reach 10% by 2035 [27].

Depending on pancreatic insulin secretory function, DM is classified as insulin-dependent type 1 diabetes mellitus (T1DM), which occurs when the pancreas produces little or no insulin, and insulin-independent type 2 diabetes mellitus (T2DM), which occurs when insulin is produced but fails to meet the body’s metabolic glucose demands. T2DM is the most common form, accounting for more than 90% of all cases worldwide.

One of the manifestations of DM is impaired lipid metabolism: elevated lipid levels are common, affect cellular metabolism, and contribute to disease progression [50]. Dysregulated lipid metabolism combined with excessive caloric intake leads to obesity, the most prevalent metabolic disorder of the endocrine system [51]. The pathophysiology of metabolic diseases is highly complex. Triggers include insufficient glucose utilization due to insulin resistance, obesity, hypercortisolemia, or receptor-mediated inhibition of serine/threonine kinases such as AKT [52]. T2DM is one of these metabolic disorders [51].

Drug-induced diabetes has become an increasingly serious issue in clinical practice and is currently recognized as a component of secondary diabetes. Pharmacological agents exacerbate this problem in populations already at high risk for glucose metabolism disturbances due to unhealthy lifestyles and the high prevalence of overweight and obesity [52]. Commonly used drugs in clinical practice can disrupt glucose homeostasis and provoke impaired glucose tolerance, hyperglycemia, or newly diagnosed diabetes, as well as worsen glycemic control in patients with pre-existing diabetes [25].

Environmental chemical pollutants are also recognized as contributing factors to the development of DM [27]. Obesity induced by environmental toxins, insulin resistance, and diabetes development are mediated by the ligand-activated transcription factor (AhR), which functions as a xenobiotic sensor. Drug-induced disruption of glucose homeostasis may also involve AhR, for which drugs act as ligands [52,53].

## 3. AhR

### 3.1. Structure, Main Functions, and Signaling Pathway of AhR

AhR is a member of the basic helix-loop-helix Per-ARNT-Sim family of transcription factors. Members of this family are involved in gene expression networks that regulate numerous physiological and developmental processes, including responses to environmental signals [54,55,56,57]. 

The human AhR structure comprises three functional domains arranged from the amino (N-) to the carboxy (C-) terminus. These include the bHLH domain, required for DNA binding; two tandemly arranged PAS (Per-ARNT-Sim) domains, PAS A and PAS B, involved in ligand binding and protein–protein interactions; and transcriptional activation domains (TADs). Activity of TADs is mediated by coactivators such as CBP/p300 and RIP140 [55,58,59]

Traditionally, AhR has been recognized as a biological sensor of planar chemical AhR is generally considered to be a factor activated by chemical compounds with a planar structure of both exogenous and endogenous origin [60,61,62], which underlies its established role in xenobiotic metabolism, particularly environmental pollutants. For a long time, AhR attracted attention mainly from toxicologists studying its role in the metabolism of 2,3,7,8-tetrachlorodibenzodioxin (TCDD) and similar compounds, PAHs, and polychlorinated biphenyls (PCBs), resulting in hyperactivation of target gene transcription and release of toxic and carcinogenic compounds [63,64,65,66]. Subsequent research has demonstrated that AhR is activated not only by planar xenobiotics but also by numerous low-molecular-weight endogenous and exogenous compounds [67,68,69].

The physiological role of AhR was established in the regulation of such processes as angiogenesis [70,71], hematopoiesis [72], cell motility [73], oncogenesis [64], immunity [67,74], and drug metabolism [75]. AhR participates in the control of cellular proliferation and the cell cycle, differentiation and phenotyping, as well as cell adhesion and migration [76,77,78,79,80,81].

**Figure 2:**
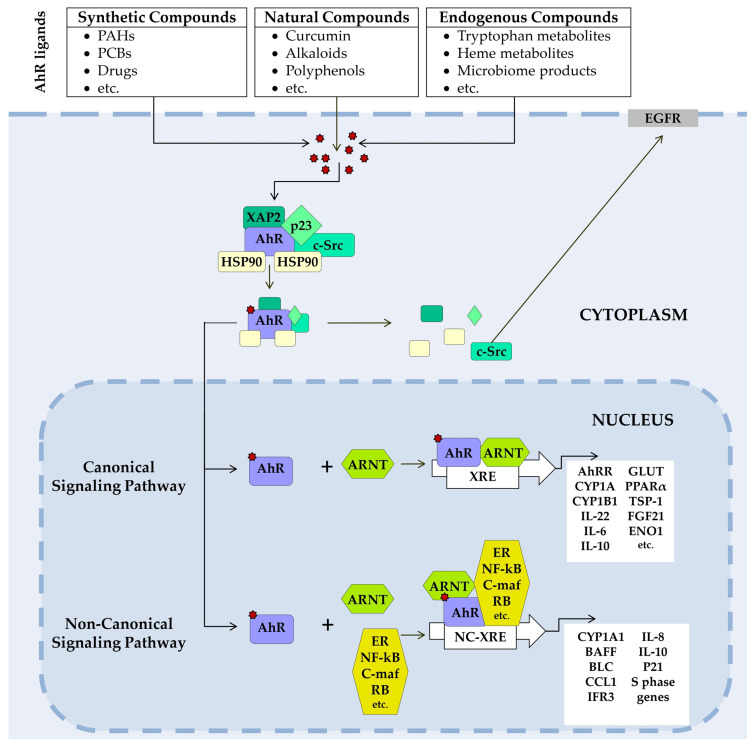
AhR signaling pathways. In its unliganded state, AhR is located in the cytosol in a complex with two HSP90s, chaperones (XAP-2), the cochaperone p23, and other partners, as well as the tyrosine kinase c-Src. A ligand-binding changes AhR’s conformation, the complex dissociates, and AhR translocates to the nucleus, where it forms a dimer with ARNT (canonical pathway) or (non-canonical pathway) with non-ARNT partner proteins such as KLF6, NF-κB, pRb, or nuclear receptors (e.g., ERα). The dimer binds to XRE or NC-XRE in DNA and induces transcription of AhR target genes. In nongenomic signaling AhR dissociated from the c-Src complex and AhR can interact with epidermal growth factor receptor (EGFR) involved in focal adhesion kinase FAK pathway and mitogen-activated protein kinase (MAPK) pathways. ARNT, aryl hydrocarbon receptor nuclear translocator; CYP, cytochrome P450; ENO1, alpha-enolase; ERα, Estrogen receptor alpha; Fgf21, fibroblast growth factor 21; GLUT, glucose transporter; HSP90, heat shock proteins 90; KLF6, transcription factor Krüppel-like factor 6; NC-XRE, nonconsensus XRE; NF-κB, transcription factors of the nuclear factor kappa B family; PPARα, peroxisome proliferator-activated receptor α; pRb, retinoblastoma protein; XAP-2, hepatitis B virus X-associated protein 2; XRE, a xenobiotic-responsive element.

AhR signal transduction (Figure 2) occurs via canonical and noncanonical pathways [62,82].

At rest, AhR resides in the cytosol in a complex with specific chaperone proteins—two HSP90, hepatitis B virus X-associated protein 2 (XAP2, also known as AIP or ARA9), p23, and c-Src tyrosine kinase [82,83,84,85]. After ligand binding, the chaperone complex dissociates, and the AhR-ligand complex translocates into the nucleus [86,87].

In the canonical pathway, the AhR–ligand complex heterodimerizes with the aryl hydrocarbon receptor nuclear translocator (ARNT). The resulting AhR/ARNT dimer binds to xenobiotic response elements (XREs) on DNA, initiating transcription of a range of genes, including members of the cytochrome P450 (CYP) 1 subfamily A and B (CYP1A1, CYP1A2, CYP1B1). Activation of these genes produces a broad spectrum of physiological and toxic effects [84,88,89,90,91].

In noncanonical pathways, the AhR–ligand complex heterodimerizes not with ARNT but with other protein partners. These are RelB and Krüppel factor 6 (KLF6) [92,93].

In addition, AhR is involved in nongenomic signaling. Upon dissociation from the c-Src complex, AhR can interact with the epidermal growth factor receptor (EGFR). The downstream signaling pathway of EGFR includes the focal adhesion kinase FAK pathway and the MAPK pathways called RAS–RAF–MEK1/2–ERK1/2 and AKT–PI3K–mTOR, as well as protein kinase C (PKC), SRC proteins, STAT, and NF-κB [94,95,96,97].

Additionally, AhR can interact with other signal transduction pathways [92,98,99,100,101,102,103,104].

### 3.2. Target Genes and AhR Ligands

AhR target genes include xenobiotic biotransformation enzymes, encompassing phase I enzymes CYP1A1 and CYP1A2 and CYP1B1; phase II enzymes such as NADPH:quinone oxidoreductase (NQO1), UDP-glucuronosyltransferases (UGT) 1A1 and 1A6, glutathione S-transferase (GST) A2, and aldehyde dehydrogenase (ALDH) 3A1 [55,74,105,106,107,108]. 

After ligand binding, AhR-dependent gene transcription may be either upregulated or downregulated depending on cellular conditions or cell type. Different cell types may exhibit distinct transcriptomic profiles in response to AhR activation, determining the physiological outcome [109,110,111].

Typical exogenous AhR ligands include halogenated aromatic hydrocarbons [112], polychlorinated biphenyls, and PAHs [63,64,112], as well as plant- and vegetable-derived compounds such as flavonoids, resveratrol, luteolin, and genistein [113]. Among low-molecular-weight endogenous ligands, tryptophan metabolites such as kynurenine [114], kynurenic acid [115], and microbiome-derived tryptophan metabolites [116,117], ultraviolet photoproduct of L-tryptophan 6-Formylindolo[3,2-*b*]carbazole (FICZ) [118], and Indolo[3,2-*b*]carbazole [114] are known. Other endogenous AhR ligands include heme degradation products bilirubin and biliverdin [119], tetrapyrroles [90], arachidonic acid metabolites [90,120,121], and the estrogen equilenin [121].

AhR antagonists block activation of the AhR by preventing it from binding to its ligands or interfering with AhR signaling pathway. They can achieve this through several mechanisms. Some AhR antagonists compete with ligands for the ligand-binding site on the AhR and prevent AhR from undergoing the conformational change for nuclear translocation. Some antagonists prevent AhR from binding to specific DNA sequences, thereby blocking its ability to regulate gene expression. Certain AhR antagonists can promote the degradation of AhR.

AhR antagonists can be classified as synthetic compounds or naturally occurring substances. Several compounds have been identified as AhR antagonists, including synthetic substances such as CH-223191, GNF-351, CB7993113, StemRegenin 1 (SR1), BAY2416964, IK-175, 3′-methoxy-4′-nitroflavone (MNF), HBU651, nilotinib, vemurafenib [122,123,124,125,126,127,128,129,130,131]. Among natural compounds AhR antagonists have also been identified, such as curcumin, resveratrol, flavonoids (apigenin, quercetin, kaempferol, luteolin), *α*-naphthoflavone (*α*-NF), indole-3-carbinol derivatives, jasmine [132,133].

Some of these agonists and antagonists are utilized as pharmaceuticals: flavonoids and polyphenols present in foods (e.g., quercetin) [134,135], omeprazole as a gastric acid-reducing agent [136], and certain planar compounds are being investigated as potential anticancer agents.

### 3.3. The Role of AhR in Glucose Metabolism

AhR role in glucose metabolism is multifaceted and affects both metabolic processes and processes such as insulin secretion, glucose uptake, and overall glucose tolerance.

Glucose can activate AhR by acting as its ligand. An AhR-binding site was identified in the glucose-responsive fragment of the thrombospondin-1 (TSP-1) promoter, and a constitutively active form of AhR induced activation of the TSP-1 gene promoter [137]. Upon activation, AhR forms a complex with other transcription factors, including early growth response protein 1 (Egr-1) and activator protein 2 (AP-2) [137].

High glucose levels activate AhR in endothelial and smooth muscle cells [137,138]. In endothelial cells, high glucose activates AhR, which leads to activation of the TSP-1 gene, a potent antiangiogenic and proatherogenic protein involved in the development of vascular complications in diabetes [137].

In vascular smooth muscle cells, the regulation is more complex and requires cooperative interaction between both proximal and distal promoter elements. This regulation depends on these promoter regions and on the formation of a protein complex between transcription factors binding to these sites. Unlike endothelial cells, where the response to glucose is controlled by a single promoter element, in smooth muscle cells, the TSP-1 gene is positively transactivated by cooperative interaction between the AhR receptor element and the GAS/ISRE activation site element. Thus, a single complex formed by the interaction of AhR and GAS/ISRE-binding proteins controls TSP-1 expression in glucose-stimulated smooth muscle cells [138].

Low glucose levels in hepatocytes act as a signal for the AhR pathway activation, potentially inducing nuclear translocation of AhR and modulating the expression of CYP1 family enzymes and NF-E2 p45-related factor (Nrf2). This suggests that under glucose-deprivation-induced stress, endogenous compounds, particularly kynurenine, may serve as AhR ligands [139]. Glucose depletion triggers endoplasmic reticulum oxidative stress, which activates the PERK-eIF2α-ATF4 stress axis, restoring intracellular glutathione pools through increased cystine and cysteine uptake and enhanced cysteine production via cystathionine γ-lyase (CSE). Additionally, low cysteine levels in endolysosomes promote tryptophan degradation to kynurenine, an endogenous AhR ligand [140].

AhR signaling is a critical regulator of pancreatic β-cell function and glucose homeostasis. In β-cells, AhR signaling maintains basal function and supports glucose and lipid homeostasis by modulating insulin secretion [141]. The study results showed that suppression of AhR in INS-1 cells disrupted normal β-cell function via multiple pathways, including reduced glucose uptake, diminished insulin production, and decreased expression of Glut2, Ins1, Ins2, and Pdx-1, without affecting cell viability or inducing apoptosis.

AhR regulates enzymes involved in glucose metabolism, including those controlling glucose transporters by modulating gene expression [142,143,144,145].

Activation of AhR can suppress the key gluconeogenic enzyme phosphoenolpyruvate carboxykinase (PEPCK), partly mediated by the AhR target gene TiPARP [144,146]. Conversely, AhR deficiency in mice is associated with elevated glucose-6-phosphatase levels, catalyzing the terminal step of gluconeogenesis [147].

AhR also influences glycolysis through metabolic reprogramming by regulating the expression of key glycolytic enzymes and glucose uptake [148]. In certain contexts, such as glioblastoma, AhR interacts with HIF-1α to control glycolysis, a hallmark of cancer cells [149]. 

AhR modulates the expression of GLUTs. AhR activation can directly enhance the activity of specific GLUTs, such as GLUT1 in human proximal tubule cells [27]. Conversely, AhR negatively regulates GLUT4 expression, impairing glucose uptake and potentially promoting insulin resistance. Environmental toxins, such as bisphenol A, decrease GLUT4 levels, limiting glucose transport into muscle and adipose tissues in response to insulin or exercise, relevant for conditions such as T2DM and polycystic ovary syndrome [150].

Thus, the AhR ligands affecting glucose homeostasis are endogenous tryptophan metabolites, which regulate positively or negatively the transcription of such target genes as genes mediating key glycolytic and gluconeogenic enzymes and genes of glucose transporter by canonical pathway and also alter the activity of mTORC1 signaling by non-genome pathway.

### 3.4. Role of AhR in Lipid Metabolism

AhR activation can modulate the expression of fatty acid synthesis genes, reducing hepatic lipogenesis in response to TCDD in both animal models and primary human hepatocytes. This effect is associated with decreased expression of key lipogenic genes, including fatty acid synthase (FASN), acetyl-CoA carboxylase-1 (ACC-1), and stearoyl-CoA desaturase-1 (SCD-1) [151].

Fatty acid translocase CD36 is recognized as a transcriptional target of AhR, as is PPARα, which mediates steatosis development [152].

AhR can inhibit mitochondrial fatty acid oxidation, negatively impacting lipid metabolism and potentially leading to dyslipidemia [153].

AhR is also implicated in the transcriptional regulation of fibroblast growth factor 21 (FGF21), a key endocrine regulator of lipid metabolism. Elevated FGF21 levels are observed in early-stage non-alcoholic fatty liver disease (NAFLD) [154]. AhR may regulate FGF21 expression depending on benz[a]pyrene dose, influencing NAFLD development [117]. FGF21 may serve as a diagnostic biomarker for metabolic disorders associated with glucose and lipid dysregulation [154].

Thus, AhR plays a certain role in lipid metabolism through its activation by environmental toxins and endogenous molecules, such as arachidonic acid metabolites and bile acids, influencing processes like fat accumulation, lipid synthesis, and breakdown. After activation, AhR influences the transcription of genes involved in lipid metabolism. Examples include the regulation of enzymes involved in fatty acid and cholesterol metabolism [155,156,157].

### 3.5. Role of AhR in Insulin Resistance

AhR plays a complex role in insulin resistance. Its involvement in the regulation of proinflammatory and metabolic processes can promote the development of insulin resistance and the progression of diabetes.

Elevated AhR expression correlates with higher levels of inflammatory markers and increased insulin resistance [158,159,160].

Environmental AhR ligands may disrupt glucose homeostasis and β-cell function through AhR activation in β-cells, potentially increasing diabetes susceptibility. AhR signaling in β-cells is essential for maintaining β-cell function in female mice and body mass in male mice, whereas exposure to high TCDD doses disrupts glucose homeostasis and β-cell function via AhR activation [158,159,160].

AhR signaling may have a dual role in insulin resistance and compensatory β-cell function. Peripheral blood transcript levels of AhR are significantly elevated in patients with familial hypercholesterolemia and T2DM, potentially promoting proinflammatory Th-cell differentiation in obesity and T2DM. This suggests a role for AhR in mediating interactions between metabolism and inflammatory status in obesity and T2DM [158,159,160].

AhR activation affects circadian rhythm regulation and gene expression which regulates metabolic processes like glucose and fatty acid metabolism. AhR activation can disrupt these rhythms, contributing to metabolic dysfunction [161]. Disruption of circadian rhythm, peroxisome proliferator-activated receptor α (PPARα) signaling (a contributor to insulin resistance and T2DM), and AhR activation are implicated in T2DM development in individuals exposed to environmental pollutants [162].

Disruption of BMAL1 and CLOCK genes alters glucose tolerance and regulation of key metabolic genes [163,164,165]. PPARα exhibits circadian oscillations, which are interdependent with BMAL1 regulation [162,166]. PPARα activation increases PEPCK and G6Pase expression, leading to hyperglycemia and insulin resistance [167].

AhR knockout mice show improved insulin sensitivity and glucose tolerance, accompanied by reduced expression of PPARα and genes encoding gluconeogenic and fatty acid oxidation enzymes [162]. Thus, PPARα activation in the liver provides a mechanism underlying AhR-mediated insulin resistance, while AhR deficiency protects mice from insulin resistance, potentially via PPARα downregulation [162].

AhR deficiency protects AhR−/− and AhR+/− mice from high-fat diet (HFD)-induced obesity, hepatic steatosis, insulin resistance, and inflammation, while preserving insulin signaling in major metabolic tissues [168]. Additionally, HFD-fed AhR−/− and AhR+/− mice exhibited reduced transcripts of key proinflammatory genes and increased anti-inflammatory IL-10 transcripts [168]. This protective effect may involve increased energy expenditure, possibly through induction of thermogenic genes in brown adipose tissue [168], as well as enhanced anti-inflammatory processes [168].

AhR activation in the liver induces the endocrine hormone FGF21, which protects against obesity and insulin resistance, uncoupling hepatic steatosis from insulin resistance [169]. FGF21, primarily produced in the liver [170], is induced via PPARα activation [171,172] and identified as a direct transcriptional target of AhR [169].

Alterations in gut microbiota composition, increased intestinal permeability, and consequent metabolic endotoxemia are also considered factors in T2DM development [173,174]. Bacterial products reaching visceral adipose tissue and liver trigger inflammation, promoting insulin resistance [173,174]. AhR plays a key role in maintaining gut homeostasis, as some microbiota-derived metabolites serve as AhR ligands [160]. In metabolic syndrome, production of AhR agonists by gut microbiota is reduced [175,176]. Restoration of AhR signaling mitigates metabolic disturbances, including glucose metabolism dysregulation and hepatic steatosis [169,175,176].

AhR ligands such as tryptophan, indole-3-carbinol, and indigo protect against insulin resistance in diabetes model systems [177].

In the Ins2Akita mouse model of insulin-dependent diabetes, tryptophan and indole-3-carbinol alleviate diabetes-induced intestinal barrier dysfunction, insulin resistance, systemic inflammation and FMO3/ICAM expression [177]. Indole-3-carbinol also stimulates glucagon-like peptide-1 (GLP-1) secretion, reducing insulin resistance and ameliorating T2DM symptoms [175]. Tryptophan is metabolized by gut microbiota into 5-hydroxyindole-3-acetic acid, which promotes ubiquitin-proteasome degradation of Suv39h1, TSC2 expression, and inhibits mTORC1 signaling, ultimately enhancing insulin signaling and reducing T2DM risk [178].

In HFD-induced obesity mouse models, the natural anti-inflammatory AhR ligand indigo effectively protects against glucose dysregulation [160]. Indigo supplementation increased Lactobacillus abundance in the gut, stimulated IL-22 production, protected pancreatic β-cells from inflammation, reversed hyperglycemia-induced damage, and improved insulin sensitivity [179]. Indigo treatment also elevated IL-10 production by liver and visceral adipose tissue immune cells [160]. IL-10, an anti-inflammatory cytokine, suppresses inflammatory mediator production and release, mitigating inflammation [180]. IL-10 stimulation reduces hyperglycemia and insulin resistance, prevents T2DM development [181], and restores gut barrier integrity and microbiome composition in HFD-fed mice [182]. AhR enhances IL-10 levels via the Src-STAT3 signaling pathway, suppressing proinflammatory macrophage phenotypes [157].

In summary, AhR signaling pathway is linked to insulin resistance through several mechanisms, including its influence on PPAR-α, circadian rhythms and inflammation. Activation of AhR by environmental toxins like dioxins promotes insulin resistance, while deficiency of AhR can protect against it. AhR signaling can also be influenced by ligands, which are natural compounds in food or metabolites of gut microbiota. AhR activation can exacerbate insulin resistance by promoting proinflammatory signaling or potentially improve it by activating metabolic regulators such as FGF21. The net effect of AhR on insulin resistance appears to depend on tissue context and the nature of activating ligands.

AhR is a critical player in the regulation of glucose and insulin dynamics. However, the quantitative contribution of the AhR pathway to hyperglycemia is not fully defined, as it involves complex, multifactorial processes rather than a single, direct mechanism. Mechanisms of contribution in AhR activation by both xenobiotics (like pollutants, drugs) and endogenous ligands may be associated with impaired pancreatic β-cell function, increased oxidative stress, altered glucose and lipid metabolism, and inflammatory pathways. AhR plays a complex and context-dependent role, involving crosstalk with multiple interacting pathways related to glucose and lipid metabolism, inflammation, and oxidative stress. Key factors contributing to this complexity are also cell type specificity and hyperglycemia as activator. AhR deficiency or inhibition often improves insulin sensitivity and glucose tolerance in animal models [27,141,158,159,162,168,169,183]. The complexity of AhR signaling means that findings in one model (in vitro cell lines, specific animal knock-out models) may not directly translate to the human condition or other models, complicating overall quantification.

## 4. AhR Ligands as Pharmaceutical Agents Impairing or Preventing Glucose Metabolism Dysregulation

A wide range of pharmacological compounds may alter glucose homeostasis through diverse mechanisms: reduction in tissue insulin sensitivity via intrinsic direct mechanisms; promotion of weight gain; and/or functional impairment of insulin secretion. Some agents also enhance hepatic glucose production, induce acute pancreatitis, or exert direct cytotoxic effects on pancreatic β-cells [25].

The principal mechanisms of drug-induced hyperglycemia include diminution of insulin secretion and/or insulin production, diminution of peripheral insulin sensitivity and/or promotion of weight gain, an increase in glucose production through promotion of hepatic gluconeogenesis, and/or glycogenolysis and destruction of pancreatic cells, leading to beta-cell injury [25,184,185,186].

The exact incidence of drug-induced hyperglycemia and diabetes remains unknown; however, evidence suggests that glucocorticoid therapy, antipsychotic agents, cardiovascular drugs (statins, β-blockers, diuretics), certain anti-infective and anticancer drugs, mTOR inhibitors/immunosuppressive agents, and tyrosine kinase inhibitors are associated with disturbances in glucose metabolism and an increased risk of hyperglycemia and/or diabetes [23,184,187,188].

Several examples of AhR involvement in mechanisms underlying drug-induced diabetes were described.

### 4.1. Crosstalk Between AhR and Other Signaling Pathways

Glucocorticoids provide a classical example of such interaction. These agents are widely prescribed as potent anti-inflammatory and immunosuppressive drugs for a broad spectrum of diseases. However, their use is also associated with adverse effects, including newly diagnosed hyperglycemia in patients without a prior history of diabetes, or severe uncontrolled hyperglycemia in patients with established diabetes [189].

Although widely prescribed for their anti-inflammatory and immunosuppressive properties, glucocorticoids have a number of common metabolic side effects, including diabetes [189].

The mechanisms of glucocorticoid-induced DM include a decrease in peripheral insulin sensitivity and/or weight gain, increased glucose production due to stimulation of hepatic gluconeogenesis, destruction of pancreatic cells leading to β-cell damage and β-cell dysfunction, impaired insulin release, inhibited glyceroneogenesis, and increased fatty acid levels [189].

Glucocorticoid-induced hyperglycemia manifests both directly via glucocorticoid signaling in metabolic organs and tissues (liver, adipose tissue, muscle, bone, and pancreatic β-cells), and indirectly through inter-organ fluxes of hormones and metabolites [190].

AhR interacts with glucocorticoids in regulating glucose homeostasis. Potential crosstalk occurs at the level of steroid hormone receptors. AhR and the glucocorticoid receptor exhibit a complex reciprocal interplay, whereby they may physically interact, regulate each other’s expression levels, and synergistically activate or suppress target gene expression in a context-dependent manner. The nature of this interaction—synergistic activation versus antagonistic repression—depends on cell type and ligand specificity [191,192,193].

Such interactions may modulate systemic responses to endogenous hormonal signals, potentially leading to endocrine disruption and inflammation.

### 4.2. Pharmacological Ligands of AhR

Examples of clinically used AhR ligands include antipsychotics, omeprazole, propranolol, tamoxifen, tranilast, flutamide, leflunomide, statins and benzothiazoles.

#### 4.2.1. Clozapine

Structurally related atypical antipsychotics clozapine and olanzapine are effective in the treatment of schizophrenia with minimal extrapyramidal motor side effects; however, their endocrine and metabolic adverse effects contribute to a broad range of metabolic complications [194,195,196].

Glucose metabolism disturbances, such as hyperglycemia and insulin resistance, are among the most frequent adverse effects of antipsychotics [14,15]. Clozapine carries the highest risk of metabolic syndrome among this drug class [194].

In HepG2 cell models, clozapine directly activated AhR signaling, upregulating CYP1A1 gene and protein expression [52]. Unlike olanzapine, clozapine dose-dependently activated AhR in the liver, aorta, and preadipocytes, adversely affecting vasodilation and adipogenesis [52].

AhR ligands such as PAHs induce metabolic abnormalities, including obesity, insulin resistance, tachycardia, and hypertension [156]. Similar adverse effects of clozapine may be attributable to its role as an AhR ligand [52,53].

AhR activation contributes to adipogenesis, obesity, insulin resistance, and depletion of glucose stores [52]. Several mechanisms of metabolic changes leading to these side effects are possible [52].

Insulin resistance and depletion of glucose reserves may occur as a result of the activated AhR blocking the AKT kinase-glucose pathway. Depletion of tryptophan and glutathione reserves may also interfere with glucose uptake and increase oxidative stress, leading to cysteine deficiency and subsequent tryptophan degradation. An AhR ligand such as kynurenine may be involved in glutathione depletion [52].

AhR activation inhibits AMP-activated protein kinase (AMPK), which is the key regulator of cellular energy homeostasis and glucose uptake, thereby promoting lipid accumulation and metabolic dysfunction. AMPK inhibition leads to iron depletion, stabilization of HIF-1, and a glycolytic shift, potentially inducing mitochondrial dysfunction [52].

#### 4.2.2. Omeprazole

The relationship between omeprazole, a proton pump inhibitor (PPI), and diabetes is complex. Some studies report an increased risk of T2DM with long-term PPI use, whereas others suggest potential improvement of glycemic control in diabetic patients [197,198,199].

Chronic omeprazole therapy induces hypergastrinemia, hyperinsulinemia, and elevated pancreatic expression of Pparg, Sirt1, and Cxcl5 in mice, accompanied by specific alterations in glucose metabolism [199]. Potential mechanisms contributing to increased risk include gut microbiota alterations, magnesium deficiency, and reduced pregnane X receptor (PXR) activity [198]. A case of autoimmune insulin syndrome, characterized by elevated anti-insulin antibodies and hyperinsulinemic hypoglycemia, was reported following omeprazole administration in the absence of exogenous insulin use [200].

Conversely, omeprazole therapy has been associated with improved glycemic control in diabetic patients receiving antidiabetic agents such as metformin and glimepiride, as evidenced by reductions in blood glucose and HbA1c levels [197,201].

In db/db mice, combined omeprazole and exendin-4 treatment reduced food intake and weight gain (likely through modulation of plasma ghrelin and leptin) and improved pancreatic insulin and glucagon content via enhanced glucokinase activity [202].

The interaction of omeprazole with diabetes involves the activation of AhR. It is known that omeprazole acts as an AhR agonist [203,204,205]. Omeprazole acts as a non-classical AhR ligand via a non-canonical mechanism [206]. It is believed that omeprazole activates AhR via phosphorylation. [207].

Omeprazole-induced AhR activation reduced oxidative damage in peripheral blood mononuclear cells [204] and demonstrated protective effects against oxidative stress and diabetic retinopathy [208].

However, studies of the long-term effects of omeprazole show that AhR activation can cause metabolic changes in mice, including increased levels of insulin, gastrin, and blood glucose [199].

#### 4.2.3. Propranolol

Propranolol, a non-selective β-adrenergic blocker prescribed for the treatment of hypertension, may impair the recovery of normal glucose levels following hypoglycemia in diabetic patients by blocking adrenaline-stimulated glucose release. Although it does not directly affect diabetes, propranolol may cause hypoglycemia, thereby exacerbating its symptoms [209].

β-adrenergic stimulation enhances the secretion of insulin and glucagon, glycogenolysis, gluconeogenesis, and lipolysis. In non-diabetic individuals, β-blockers pose a minimal risk of impaired glucose regulation. In insulin-dependent diabetes, however, β-blockers may prolong, intensify, or alter the symptoms of hypoglycemia, whereas hyperglycemia appears to be the primary risk in non-insulin-dependent diabetes. β-blockers may also increase blood glucose concentrations and antagonize the action of oral hypoglycemic drugs [18,209].

It was reported that propranolol, and particularly its photoproduct formed under ultraviolet (UV) irradiation, can activate AhR and stimulate the secretion of pro-inflammatory cytokines [210]. UV exposure markedly enhanced the ability of propranolol, acting as a non-competitive AhR antagonist, to activate AhR and induced a pro-inflammatory AhR signaling pathway identical to that triggered by FICZ under UV exposure [211].

At present, no published data are available on whether propranolol and its photoproduct as AhR ligands exert differential effects on blood glucose concentrations or interfere with the efficacy of hypoglycemic drugs.

#### 4.2.4. Hydroxytamoxifen

Tamoxifen, a hormonal therapy used in the treatment and prevention of estrogen receptor–positive breast cancer, has been associated with an increased risk of T2DM, particularly in elderly women and those with pre-existing risk factors, as tamoxifen may enhance insulin resistance and exacerbate the latent risk of diabetes in predisposed women [212].

The development of hyperglycemia during tamoxifen therapy, the major metabolite of which is 4-hydroxytamoxifen (4OHT), is facilitated by impaired β-cell secretory activity associated with inhibition of calcium and anion channel currents [213].

4OHT exhibits AhR-dependent activity by directly binding to AhR and modulating its transcriptional activity. In the absence of estrogen receptors, 4OHT can induce the expression of AhR target genes involved in estradiol metabolism, cell proliferation, and metastasis in breast cancer cell models [214]. Tamoxifen also influences adipose tissue biology, where AhR expression has been detected.

The role of AhR in tamoxifen-induced insulin resistance remains unexplored [215]; however, given its metabolic effects and the established role of AhR in insulin resistance, a potential mechanistic link has been suggested [216].

#### 4.2.5. Tranilast

The antiallergic agent tranilast (N-(3,4-demethoxycinnamoyl)-anthranilic acid) is an inhibitor of mast cell degranulation. Tranilast shows potential for treating complications of diabetes. Tranilast has been shown to reduce albuminuria in a rat model of diabetic nephropathy and to reduce vascular hypertrophy in diabetic rats, suggesting its clinical efficacy in treating complications of diabetes. Treatment with tranilast suppressed glucose-induced insulin secretion in INS-1E cells and rat pancreatic islets. Tranilast inhibits tolbutamide-induced insulin secretion. Treatment with tranylcypromine enhanced glucose uptake by INS-1E cells. Tranylcypromine has been shown to inhibit glucose- and tolbutamide-induced insulin secretion by activating potassium (K(ATP)) channels in pancreatic β-cells [16].

In a study of gestational diabetes mellitus (GDM) using pregnant C57BL/KsJdb/+ db/+ mice, tranilast significantly improved GDM-related parameters, including maternal body weight, hyperglycemia, insulin deficiency, impaired glucose tolerance, and insulin resistance, as well as offspring size and reduced offspring body weight. Thus, tranilast may serve as a therapeutic option for GDM [217].

Tranilast was also shown to inhibit lipoapoptosis and restore glucose-stimulated insulin secretion under high palmitic acid exposure [218]. These effects are thought to be mediated via FoxO-1 inactivation. In vivo studies in wild-type and db/db diabetic mice demonstrated improved glucose tolerance along with FoxO-1 inactivation in pancreatic tissue following tranilast treatment [218].

Tranilast, as a tryptophan derivative, is a known AhR agonist [219,220], affecting cellular reprogramming, growth, and inflammation. It has been investigated as a therapeutic agent in breast cancer [220] and lymphoid malignancies [219]. Beyond oncology, tranilast’s AhR-mediated effects may also have implications for other immune- and inflammation-related conditions, although clinical evidence is currently lacking, and this remains an area of active investigation [94].

#### 4.2.6. Leflunomide

Leflunomide, a drug used in the treatment of rheumatoid arthritis, shows potential in diabetes therapy by enhancing insulin sensitivity and reducing hyperglycemia in mice. In diabetic ob/ob mice fed a high-fat diet, leflunomide normalized blood glucose levels and reversed insulin resistance in glucose and insulin tolerance tests [20].

Leflunomide is a direct AhR agonist, and AhR activation accounts for its anti-inflammatory properties [221]. Leflunomide acts on molecular pathways implicated in insulin resistance, a central factor in T2DM [20], pathways in which AhR signaling may also be involved.

Currently, leflunomide is not indicated for diabetes treatment due to associated risks such as hepatotoxicity and neuropathy, which are of particular concern in diabetic patients.

#### 4.2.7. Flutamide

Flutamide, an oral nonsteroidal antiandrogen used for prostate cancer treatment, is associated with idiosyncratic hepatotoxicity. Flutamide significantly reduces both fasting insulin secretion and insulin response during oral glucose tolerance testing, but only in women with idiopathic hirsutism. Treatment with flutamide may completely eliminate hyperinsulinemia in such patients, suggesting that its efficacy depends on peripheral hyperandrogenic activity [222].

Polycystic ovary syndrome (PCOS) is closely linked with metabolic syndrome and cardiometabolic risk, predisposing women to an increased risk of T2DM and cardiovascular disease. In rodent models of PCOS, treatment with 300 mg/kg metformin, 30 mg/kg flutamide, or their combination for six weeks demonstrated differential effects: metformin improved fasting insulin and HOMA-IR, while flutamide and combination therapy reduced plasma triglycerides, ApoB48, and ApoB100 [21].

Alongside leflunomide, flutamide has been identified as an AhR agonist, without causing dioxin-like toxicity in rats or humans [221]. Expression of AhR-regulated gene sets was markedly increased in the liver of wild-type Ahr+/+ mice treated with flutamide but not in Ahr−/− mice. Ligand-docking analyses predicted flutamide to be an AhR agonist, which was confirmed by luciferase reporter assays. Expression of mRNA encoding the bile acid transporter ABCC4 was elevated, while farnesoid X receptor signaling was suppressed in the liver of Ahr+/+ but not Ahr−/− mice treated with flutamide [223].

#### 4.2.8. Statins

Recent evidence indicates that statins increase the risk of newly diagnosed T2DM and, at high doses, worsen glycemic control in patients with T2DM. A systematic review of 33 publications encompassing 1 951 113 participants found that, among 20 studies investigating atorvastatin-related diabetes dysregulation, approximately half reported no significant effect on glycemic control, whereas the other half demonstrated elevated fasting plasma glucose and HbA1c levels. High-dose atorvastatin was shown to impair glycemic control in T2DM patients [19].

Since the first reports of statin-induced hyperglycemia and diabetes, cumulative evidence has supported a causal association. There is consensus that high-intensity statin therapy and patients with obesity or glycemic indices close to the diabetic threshold represent the highest-risk groups. Alterations in insulin signaling, glucose transport, and gut microbiota composition are the principal mechanistic hypotheses underlying statin-induced hyperglycemia [224].

Atorvastatin and pravastatin, cholesterol-lowering drugs, have been shown to activate AhR, inhibit lipopolysaccharide-induced inflammatory responses, and promote an AhR-dependent M2 macrophage anti-inflammatory phenotype [225].

In dextran sulfate sodium-induced ulcerative colitis in mice, atorvastatin altered tryptophan metabolism and increased fecal concentrations of tryptophan, indolelactic acid, and indole-3-acetic acid. In addition, atorvastatin increased AhR expression [226].

Conversely, studies on the effects of atorvastatin, fluvastatin, and rosuvastatin optical isomers on cytochrome P450 drug-metabolizing enzymes in primary human hepatocytes found no significant impact on AhR transcriptional activity or CYP1A1/CYP1A2 expression. Nevertheless, all statins induced CYP2A6, CYP2B6, CYP3A4, and partially CYP2C9 [227].

It was shown that atorvastatin and pravastatin, when exposed to activated macrophages, could enhance arginase expression and stimulate an anti-inflammatory response in an AhR-dependent manner [225]. Pravastatin and atorvastatin and enhanced arginase expression and anti-inflammatory response in activated macrophages in an AhR-dependent manner. These findings suggest that pravastatin and atorvastatin activate AhR, attenuate LPS-induced inflammation, and foster the M2 macrophage phenotype in an AhR-dependent manner. Collectively, these data propose AhR as a novel target for the anti-inflammatory effects of statin therapy. However, the implications of AhR inhibitors or activators on T2DM risk or glycemic profile alterations in statin-treated diabetic patients remain to be determined.

#### 4.2.9. Benzothiazoles

Benzothiazoles are bicyclic heteroaromatic compounds that function as AhR ligands [228]. They exhibit a broad spectrum of biological activities, including antidiabetic effects [229]. Several commercially available drugs containing the benzothiazole scaffold are used in the treatment of cardiovascular diseases, glaucoma, and as antimicrobial agents [230,231,232]. Zopolrestat was developed for the treatment of diabetic complications [233]. A number of studies have shown that the antidiabetic activity of benzothiazole derivatives is linked to their agonistic action on PPAR receptors [234].

The effects of pharmaceutical agents on glucose metabolism with potential involvement of AhR are summarized in Table 1.

## 5. The Role of AhR in the Mechanisms of Action of Antidiabetic Agents

There are several examples demonstrating how AhR involvement in the action of antidiabetic drugs reveals their novel therapeutic potential.

Metformin, belonging to the class of oral hypoglycemic agents, exhibits anti-inflammatory properties beyond its glucose-lowering activity. Metformin may act as an inhibitor of AhR and its downstream genes, as it reduces expression of CYP1A1 and CYP1B1 in MCF-7 breast cancer cells by suppressing the AhR signaling pathway [237]. First, metformin-mediated downregulation of CYP1A1 and CYP1B1 is unrelated to estrogen receptor signaling. Second, the observed metformin-induced reduction in AhR expression was mediated through decreased levels of the Sp1 protein. Third, in MCF-7 cells metformin inhibited endogenous ligand-induced AhR expression of CYP1B1 and CYP1A1 by suppressing tryptophan-2,3-dioxygenase (TDO) expression. Finally, metformin inhibited TDO expression, a key enzyme of the tryptophan metabolism pathway responsible for generating endogenous AhR ligands such as kynurenine, through suppression of Sp1 and glucocorticoid receptor (GR) protein levels. Thus, metformin decreases CYP1A1 and CYP1B1 expression in breast cancer cells by suppressing the AhR signaling pathway and may act as a potential chemopreventive agent against CYP1B1- and CYP1A1-mediated carcinogenesis and cancer progression [237].

Mechanistic insights into how metformin may regulate AhR-mediated mast cell functions were presented in publications, suggesting its potential therapeutic value in asthma and allergic diseases [238]. Metformin, at pharmacological concentrations, was shown to inhibit AhR-potentiated and IgE-mediated mast cell activation both in vivo and in vitro, at least in part by blocking enhanced sphingosine-1-phosphate (S1P) synthesis [238]. In addition, evidence was obtained for a novel mechanism regulating T-cell activation suppressor via AhR [239].

Metformin significantly reduced levels of the V-domain Ig suppressor of T cell activation (VISTA) and AhR both in vitro and in vivo, and inhibited all AhR-regulated genes. VISTA expression was strongly suppressed by AhR modulation using shRNA and α-naphthoflavone (αNF). Finally, metformin markedly reduced both tumor volume and growth rate in xenotransplanted melanoma. These results provide the first evidence that metformin suppresses VISTA, identify a novel regulatory mechanism via AhR, and highlight metformin as a potential new therapeutic strategy for melanoma treatment in combination with targeted immune checkpoint inhibitors [239].

The flavonoid quercetin, abundant in many fruits and vegetables, has demonstrated antidiabetic properties in vivo and in vitro. Quercetin improves glucose tolerance and pancreatic β-cell insulin secretion. It inhibits DPP-IV enzymes and α-glucosidase, thereby prolonging glucagon-like peptide-1 (GLP-1) half-life and glucose-dependent insulinotropic polypeptide (GIP) [240].

Quercetin showed effects comparable to those of the antidiabetic drug metformin in cellular models [241,242]. In vivo studies also demonstrated its promise as a therapeutic agent for diabetes and its pathophysiological complications. Quercetin stimulates insulin secretion, protects pancreatic β-cells from reactive oxygen species, and enhances cellular antioxidant defense mechanisms [243,244].

Thus, quercetin may represent a promising agent for T2DM treatment, potentially lowering blood glucose, improving insulin sensitivity, and reducing oxidative stress [245]. Importantly, quercetin is also an AhR ligand [113,135]. Quercetin at least partially attenuates BaP-induced CYP1A expression by inhibiting AhR functional activity and enhancing the transcriptional activity of negative regulators of AhR-sensitive genes, such as C/EBPβ and Oct-1 [246].

## 6. Conclusions

The issue of drug-induced DM is of significant clinical relevance, particularly for individuals predisposed to glucose metabolism disorders due to genetic factors or unhealthy lifestyles. Pharmacological agents can alter glucose homeostasis and provoke impaired glucose tolerance, hyperglycemia, or newly diagnosed diabetes mellitus, as well as worsen glycemic control in patients with preexisting diabetes. Impaired glucose metabolism is a key trigger for diseases associated with metabolic syndrome.

In recent years, AhR has been identified as an important modulator of various diseases. Exposure to xenobiotic AhR ligands, particularly environmental pollutants, may contribute to the rising incidence of metabolic disorders, including DM. Moreover, increased AhR expression and activity mediated by other exogenous and endogenous ligands can exert multifaceted effects on the pathophysiology of metabolic abnormalities, including diabetes. AhR represents a convergence point for signaling pathways that convey information about both extracellular and intracellular environments. A growing body of evidence demonstrates that AhR plays a key role in glucose signaling and metabolism. Its activation by glucose and other compounds is intricately linked with insulin secretion, glucose uptake, and the development of metabolic disturbances. AhR signaling is a critical regulator of pancreatic β-cell function. It also regulates the expression of enzymes involved in glucose metabolism, including those controlling glucose transporters. AhR can influence glycolysis through metabolic reprogramming, modulating the expression of key glycolytic enzymes and affecting glucose uptake. Both canonical and noncanonical AhR signaling may be implicated in mechanisms including altered insulin secretion and sensitivity, increased glucose production, and direct cytotoxic effects on pancreatic cells. In glioblastoma, AhR may interact with HIF-1α to control glycolysis, a hallmark of cancer cell metabolism. AhR negatively regulates GLUT4 expression, leading to impaired glucose uptake and potential insulin resistance. AhR also plays an important role in lipid metabolism, influencing fatty acid synthesis and oxidation, and regulating FGF21, a key factor in lipid homeostasis. The role of AhR in insulin resistance is complex, as it participates in the regulation of both metabolic and immune processes, disturbances of which may promote the development of insulin resistance. AhR activation may either exacerbate insulin resistance through the induction of proinflammatory signaling or potentially ameliorate it via activation of metabolic regulators such as FGF21.

Given the diversity of AhR ligands, it is unlikely that all ligands act uniformly; thus, a careful comparison of their effects on metabolic pathways is warranted. Overall, the precise effect of AhR on insulin resistance appears to depend on factors such as tissue type and the specific ligands activating AhR, with AhR deficiency generally exerting a protective effect against insulin resistance. Thus, quantifying the exact contribution of the AhR pathway to overall hyperglycemia in humans is challenging to determine due to its complex and context-dependent role in multiple interacting pathways.

In this review, we summarize findings that highlight the role of AhR in drug-induced glucose metabolism dysregulation. Pharmacological compounds may alter glucose homeostasis through various mechanisms, including decreased insulin sensitivity via direct intracellular pathways, promotion of weight gain, and/or functional impairment of insulin secretion—potentially involving AhR.

This applies to certain existing drugs such as glucocorticoids and estrogens, which appear to directly or indirectly affect the AhR signaling pathway. Some medications known to induce glucose metabolism disorders act as AhR ligands. Experimental studies have shown that insulin resistance and glucose depletion may occur through AhR activation leading to AKT kinase–glucose pathway blockade (as in the case of the antipsychotic drug clozapine) or via AhR-mediated induction of proinflammatory signaling (as seen with the nonselective β-blocker propranolol). A more complex relationship is observed between DM and omeprazole, a proton pump inhibitor acting as a noncanonical AhR ligand. Omeprazole-induced AhR activation can help prevent diabetic retinopathy; however, long-term exposure may lead to metabolic alterations, including increased insulin and blood glucose levels. Tamoxifen, used in hormone therapy, can increase insulin resistance and exacerbate latent diabetes risk in predisposed women. Its active metabolite, 4-hydroxytamoxifen, binds to AhR and modulates its transcriptional activity. The metabolic effects of tamoxifen and its known role in insulin resistance suggest a potential link between AhR activation and tamoxifen-induced insulin resistance, although this connection remains to be fully elucidated. Statins such as atorvastatin and pravastatin may activate AhR and promote an AhR-dependent anti-inflammatory M2 macrophage phenotype. Based on these findings, AhR has been proposed as a novel target mediating the anti-inflammatory effects of statin therapy. However, the implications of AhR inhibitors or activators for DM risk and glycemic control in statin-treated patients require further investigation.

Conversely, some AhR ligands may prevent glucose metabolism dysregulation. The anti-allergic drug tranilast, a tryptophan derivative and AhR agonist, has shown potential in the treatment of diabetic complications—an area currently under investigation. The antirheumatic drug leflunomide, also an AhR agonist, demonstrates potential antidiabetic effects by enhancing insulin sensitivity and reducing hyperglycemia, likely mediated through its AhR-dependent anti-inflammatory properties. Another AhR agonist, flutamide, a nonsteroidal antiandrogen, has been shown to reduce hyperinsulinemia. The antidiabetic agent zopolrestat, containing a benzothiazole moiety, has been developed for treating diabetic complications. Benzothiazoles, a class of bicyclic heteroaromatic compounds, are known AhR ligands.

AhR antagonists are not widely studied in relation to glucose metabolism. Drug metformin and the flavonoid quercetin are AhR antagonists, both possessing antidiabetic properties. AhR antagonists should be explored as a potential therapeutic strategy in diabetes due to their ability to reduce inflammation, improve insulin sensitivity, and mitigate obesity-associated metabolic dysfunction [131,247,248]. As for metformin and quercetin, they exemplify how AhR involvement in their mechanisms of action unveils new therapeutic potential.

AhR activation can be both a primary driver of specific drug effects (especially toxic ones) and a minor contributing factor compared to other mechanisms for these drugs in the context of broader physiological processes, depending on the specific ligand, tissue, and biological context. It is not a single, universal mechanism for all drugs and AhR acts as a complex modulator among many other biological pathways. For pharmaceuticals, the effect of AhR activation is highly variable. Some new drugs, such as tapinarof for psoriasis, are designed to target AhR as their primary therapeutic mechanism for modulating the immune response [249,250]. For many other drugs, including those described in this review, AhR activation may be a secondary effect and a minor contributor to their overall action.

In conclusion, further studies are required to elucidate the complex interplay between AhR, insulin signaling, and glucose metabolism, and to clarify the precise mechanisms through which this interaction is mediated, as current research in this area is still ongoing.

## Figures and Tables

**Table 1:** Effect of pharmaceutical agents on glucose metabolism with potential AhR involvement.

Drugs	Therapeutic Class	Role in AhR Signaling	Drug-Induced Metabolic Changes with Possible Involvement of AhR Signaling	References
Glucocorticoid	Anti-inflammatory, immune- suppressant	Cross-talk	Development of hxyperglycemia, insulin resistance and type 2 diabetes. Destruction of pancreatic cells	[189,191,192,193]
Clozapine	Atypical antipsychotics	Agonist	Hyperglycemia and insulin resistance	[14,15,52]
Omeprazole	Proton pump inhibitor	Agonist, non-classic ligand (non-classic AhR signaling)	Insulin resistance. Increased risk of T2DM with long-term use, whereas potential improvement in glycemic control in diabetic patients receiving antidiabetic agents	[197,198,199,200,205,206,207]
Propranolol	β-blocker	Agonist	Development of hypoglycemia in patients with diabetes. Impaired glucose level recovery following hypoglycemia in diabetic patients by blocking adrenaline-stimulated glucose release	[209,210,211]
Hydroxytamoxifen (Tamoxifen metabolite)	Selective estrogen receptor modulator	Agonist	Impaired β-cell secretory activity. Enhancement of insulin resistance and exacerbation of the latent risk of diabetes in predisposed women	[212,213,215]
Ttranilast	Antiallergic	Agonist	Enhancement of glucose uptake by INS-1E cells and suppression of glucose-induced insulin secretion in INS-1E cells and rat pancreatic islets.	[16,219,220]
Leflunomide	Antirheumatic agent	Agonist	Enhancing insulin sensitivity and reducing hyperglycemia in diabetic mice fed a high-fat diet	[20,221]
Flutamide	Antiandrogen	Agonist, selective AhR modulator	Reduced hyperinsulinemia in women with polycystic ovary syndrome but worsened glucose intolerance with high-fat diet in experimental animals	[222,223,235]
Statins	HMG-CoA reductase inhibitors	Agonist	Insulin resistance, increasing glucose production by upregulating enzymes involved in gluconeogenesis. Increased risk of developing T2DM in patients with obesity, worsening glycemic control in patients with T2DM	[19,225,236]

## Data Availability

No new data were created or analyzed in this study.

## Abbreviations

The following abbreviations are used in this manuscript:

4OHT	4-hydroxytamoxifen
AhR	Aryl hydrocarbon receptor
AKT	Protein kinase B
AMPK	AMP-activated protein kinase
ARNT	Aryl hydrocarbon receptor nuclear translocator
CYP	Cytochrome P450
DM	Diabetes mellitus
EGFR	Epidermal growth factor receptor
FGF21	Fibroblast growth factor 21
FICZ	6-Formylindolo[3,2-*b*]carbazole
GDM	Gestational diabetes mellitus
GIP	glucose-dependent insulinotropic polypeptide
GLP-1	Glucagon-like peptide-1
GLUTs	Sodium-independent glucose transporters
GR	Glucocorticoid receptor
HIF-1	Hypoxia-inducible factor-1
IRs	Insulin receptors
IRS	Insulin receptor substrates
MAPK	Mitogen-activated protein kinases
mTORC1	Mammalian target of rapamycin complex 1
NAFLD	Non-alcoholic fatty liver disease
Nrf2	NF-E2 p45-related factor
PAHs	Polycyclic aromatic hydrocarbons
PCOS	Polycystic ovary syndrome
PI3K	Phosphoinositide-3-kinase
PKC	Protein kinase C
PPARα	Peroxisome proliferator-activated receptor α
PPI	Proton pump inhibitor
PXR	Pregnane X receptor
S1P	Sphingosine-1-phosphate
SGLTs	Sodium-dependent glucose transporters
T1DM	Type 1 diabetes mellitus
T2DM	Type 2 diabetes mellitus
TADs	Transcriptional activation domains
TCDD	2,3,7,8-tetrachlorodibenzodioxin
TDO	Tryptophan-2,3-dioxygenase
TSP-1	Thrombospondin-1
UV	Ultraviolet
VISTA	V-domain Ig suppressor of T cell activation
XREs	Xenobiotic response elements
